# Identification of ISG15 and ZFP36 as novel hypoxia- and immune-related gene signatures contributing to a new perspective for the treatment of prostate cancer by bioinformatics and experimental verification

**DOI:** 10.1186/s12967-022-03398-4

**Published:** 2022-05-10

**Authors:** Fang Lyu, Yunxue Li, Zhecheng Yan, Qingliu He, Lulin Cheng, Pu Zhang, Bing Liu, Chunyu Liu, Yarong Song, Yifei Xing

**Affiliations:** 1 Department of Urology, Union Hospital, Tongji Medical College, Huazhong University of Science and Technology, Wuhan, 430022 China; 2 Department of Pathology, Union Hospital, Tongji Medical College, Huazhong University of Science and Technology, Wuhan, 430022 China

**Keywords:** Bioinformatics, Prostate cancer, Hypoxia-related genes, Immune-related genes, ISG15, ZFP36

## Abstract

**Background:**

Prostatic cancer (PCa) is one of the most common malignant tumors in men worldwide. Emerging evidence indicates significance of hypoxia and immunity in PCa invasion and metastasis. This study aimed to develop a hypoxia- and immune-related gene risk signature and explore the molecular mechanisms to formulate a better prognostic tool for PCa patients.

**Methods:**

The hypoxia and immune scores of all PCa patients in The Cancer Genome Atlas (TCGA) dataset were calculated via the maximally selected rank statistics method and the ESTIMATE algorithm. From common genes identified overlapping hypoxia- and immune-related differentially expressed genes (DE-HRGs and DE-IRGs), a hypoxia- and immune-related gene risk signature was developed utilizing univariate and multivariate Cox regression analyses, and validated in the Memorial Sloan Kettering Cancer Centre (MSKCC) database. The immune cell infiltration level of PCa samples were evaluated with ssGSEA algorithm. Differential expression of prognostic genes was evidenced by immunohistochemistry and western blot (WB) in paired PCa samples. Expression levels of these genes and their variations under regular and hypoxic conditions were examined in cell lines. The functional effects of the prognostic gene on PCa cells were examined by wound healing and transwell assays.

**Results:**

A hypoxia- and immune-related gene risk signature constructed by ISG15 and ZFP36 displays significant predictive potency, with higher risk score representing worse survival. A nomogram based on independent prognostic factors including the risk score and Gleason score exhibited excellent clinical value in the survival prediction of PCa. Infiltration levels of eosinophils, neutrophils, Tcm, Tem, TFH, Th1 cells, and Th17 cells were significantly lower in the high-risk group. Conversely, aDC, pDC, T helper cells, and Tregs were significantly higher. Additionally, the two prognostic genes were closely correlated with the tumor-infiltrating immune cell subset in PCa progression. RT-qPCR and WB presented higher and lower expression of ISG15 and ZFP36 in PCa cells, respectively. They were correspondingly increased and decreased in PCa cells under hypoxic conditions. Wound healing and transwell assays showed that over-expression of ISG15 promoted the migration and invasion of PCa cells.

**Conclusion:**

Our study identified a novel hypoxia- and immune-related gene signature, contributing a new perspective to the treatment of PCa

**Supplementary Information:**

The online version contains supplementary material available at 10.1186/s12967-022-03398-4.

## Introduction

Prostate cancer (PCa) is one of the most common malignancies worldwide, with the second highest incidence rate among men, and the fifth highest mortality rate [1, 2]. Risk factors include age, race, genetics, hormonal disorders, inflammation, and dietary habits [3–6]. Currently, there are a variety of treatment options for prostate cancer with varying results. Although not all prostate cancer is lethal, some subtypes can trigger a terrible prognosis, such as cribriform morphology—a Gleason pattern 4 subtype—recognized as an aggressive and lethal pattern of prostate cancer [7]. Thus, new biomarkers with higher predictive value need to be further explored to ascertain extent of aggressiveness and improve the prognosis of patients.

Hypoxia, a vital feature of the tumor microenvironment, is associated with tumor resistance to chemotherapy, radiotherapy, and patient survival. This suggests that hypoxia may lead to tumor progression and treatment resistance [8, 9]. Recent findings have demonstrated that hypoxia can promote PCa aggressiveness and correlates with poor prognosis [8, 10, 11]. Moreover, another study showed that hypoxia was an independent predictor of biochemical recurrence of PCa after radiotherapy alone, or in radiotherapy in combination with neoadjuvant and concurrent hormonal therapy [12]. In addition, the relationship between tumor metastasis and exosomes secreted by PCa cells in anoxic environments has also been reported [13]. Deep et al. [13] found that exosomes secreted by PCa cells under hypoxia remodelled distant pre-metastatic niches (PMN), by increasing the expression of matrix metalloproteinases (MMPs), fibronectin, collagen IV, and the number of CD11b+ cells at select PMN sites. It is evident that early detection of PMN sites before metastasis could provide a therapeutic advantage. Immune cells, known to be important in tumor suppression, are recruited to tumors from the oxygen-rich bloodstream and experience a shift to a hypoxic environment [14, 15]. However, immune cells may be functionally inhibited in the hypoxic zones. For example, the activity of hypoxia-inducible factor (HIF) in the interaction between myeloid-derived suppressor cell and T cells has been demonstrated to play a key role in tumor microenvironment immunity [15]. Hypoxia and immune infiltration also have an interesting correlation with PCa. Jayaprakash et al. [16] reported that the hypoxic zones of PCa lacked substantial infiltration of any type of T cells. They found that hypoxia acted both, directly and indirectly, to promote dysregulated tumor angiogenesis by poorly expressing the adhesion molecules necessary to support T cell extravasation. There is growing evidence demonstrating a direct or indirect interaction between hypoxia and immune status in the development of PCa, although the mechanisms are not well understood [16, 17]. Moreover, it is unclear whether hypoxia and immune-related genes are associated with prognosis in patients with PCa.

This study proposes the development of a combined hypoxia and immune gene marker to provide a better prognostic tool for PCa patients.

## Materials and methods

### Data source

A total of 532 samples were collected from The Cancer Genome Atlas (TCGA) database (V27.0, December 31, 2020), including 481 PCa tumor samples and 51 normal prostate tissue samples. Among the PCa tumor samples, 475 samples contained transcriptome data and complete survival information for calculating hypoxia and immune scores. Only 404 samples contained transcriptomic data, complete survival and clinical information (Gleason score, pathological N stage, pathological T stage, age, etc.) were used to construct a risk model. A total of 195 PCa tumor samples were obtained from the Memorial Sloan Kettering Cancer Center (MSKCC), of which 138 with complete survival and clinical information were used for further validation.

### Calculation of the hypoxia score and immune score

Hypoxia scores which represented the hypoxia status of each sample were calculated based on the maximally selected rank statistics method by using the survminer package. According to the optimal cut-off value of hypoxia score, a total of 475 PCa patients with complete survival data were assigned to high- (n = 207) and low-hypoxia score (n = 268) groups.

Immune scores were calculated by employing the ESTIMATE algorithm to the matrix data 475 PCa patients [18]. Furthermore, the 475 PCa cases were divided into high- (n = 196) and low-immune score (n = 279) groups based on the optimal cut-off value of immune scores. In addition, Wilcox test was used to assess the correlation between clinicopathological factors and hypoxia score and immune score.

### Differentially expressed analysis

The limma package was utilized to screen the differentially expressed genes (DEGs) of the high-hypoxia and low-hypoxia score groups. Similarly, the DEGs between the high-immune score and low-immune score groups were identified by the same method. The |log_2_FC|> 0.5 and false discovery rate (FDR) < 0.05 were selected as screening conditions. Hypoxia- and immune-related genes were obtained by overlapping the hypoxia-related DEGs (DE-HRGs) with immune-related DEGs (DE-IRGs).

### Functional enrichment analysis

To further explore the gene function in PCa, we performed a functional enrichment analysis based on Kyoto Encyclopedia of Genes and Genomes (KEGG) and Gene Ontology (GO) by ‘clusterProfiler’ package. GO mainly described biological process (BP), molecular function (MF) and cellular component (CC). The terms with *P-*value < 0.05 were regarded as statistically significant.

### Development and validation of a risk signature

404 PCa patients of TCGA database containing DFS information and clinical features was considered as a training set. The univariate Cox regression analysis was carried out in the training set to sort hypoxia- and immune-related genes in ascending order by their *P* values. Hypoxia- and immune-related genes with significant prognostic value (*P* < 0.05) were filtered in stepwise multivariate regression analysis to construct a risk signature. The risk score of each PCa patient in the training set was calculated by using the ‘Predict.coxph’ function in the ‘Survival’ R package. The formula is as follows: Riskscore = h0(t)*exp(β1X1 + β2X2 + … + βnXn), where β refers to the regression coefficient, and ho(t) ref the benchmark risk function. Then, the 404 PCa patients were classified to high-risk and low-risk groups based on the median of risk score. Kaplan–Meier (K–M) survival analysis with the log-rank method was employed to estimate the prognostic difference of the two risk groups. Receiver operating characteristic (ROC) analyses were executed by utilizing ‘survivalROC’ package in R. The risk signature constructed in the training set was validated in MSKCC database.

### Gene set enrichment analysis (GSEA)

To further explore the pathways related to prognostic genes in PCa, we calculated the correlation between prognostic genes and all other genes in the TCGA dataset. An ordered list of all genes was generated based on the correlation analysis with the expression of prognostic genes. Subsequently, GSEA was constructed to analyze the differences between high and low expression groups. The terms with *P* value < 0.05 and normalized enrichment score |NES|≥ 0 were considered as significant enrichment.

### Single sample gene set enrichment analysis (ssGSEA)

ssGSEA defines a enrichment score for representing the absolute enrichment degree of a particular gene set in each sample [19]. Based on previous studies, we screened 24 immune cells associated with human cancer, then downloaded 24 immune cell gene sets from the GSEA database (MigsDB), and quantified the enrichment scores of 24 immune cell gene sets using ssGSEA algorithm in the ‘gsva’ R package [20, 21]. The differences of immune infiltration level between high- and low-risk groups were analyzed by Wilcoxon test. Pearson correlation analysis was employed to investigate the relationship between prognostic genes and infiltrating immune cells.

### Statistical analysis

All statistical analyses were carried out with R (version 3.6.0). The differences in clinical features of high-risk and low-risk groups were analyzed by chi-square test in the training set and validation set, respectively. Univariate and multivariate Cox regression analyses were performed to identify independent predictors for the prognosis of PCa. A nomogram integrating a variety of independent prognostic predictors and the risk model was constructed using the ‘rms’ package in R. The expression levels of prognostic hypoxia-immune genes between tumor samples and normal samples were compared by t-test. Differences of a *P-*value < 0.05 was considered statistically significant.

### Immunohistochemistry

Samples were paraffinized, rehydrated, and blocked with 3% of H2O2, followed by incubation with normal goat serum (Vector Laboratories, Burlingame, CA, USA). After incubation with the primary antibody specific for ISG15 (1:200; Catalogue No: 15981-1-AP; ProteinTech) and ZFP36 (1:1000; Catalogue No: 12737-1-AP; ProteinTech) overnight at 4 °C, sections were washed with PBS and incubated with the biotinylated secondary antibody (Vector Laboratories), followed by incubation with Vectastain ABC Reagent (Vector Laboratories). The visualization signals were developed using diaminobenzidine (DAB, Vector Laboratories), and the slides were counterstained with hematoxylin. The images were captured using an Olympus BX60 microscope (Olympus, Japan). Immunoreactivity was scored based on a combination of both the percentage and intensity of positively stained tumor cells to generate an H-score. Staining intensity was divided into four categories as follows: no staining, 0; weak staining, 1; moderate staining, 2; strong staining, 3. H-score was determined according to the formula: (% of weak staining × 1).

### Cell culture

LNCaP, PC-3 and 22RV-1 were obtained from Shanghai Cell Bank, Chinese Academy of Sciences (Shanghai, China).RWPE-1 were provided by Dr. Jiamin Gu (Zhongnan Hospital, Wuhan, China). Cells were maintained in RPMI 1640 medium (Hyclone, GE Healthcare Life Sciences, Logan, UT, USA) with 10% foetal bovine serum (Biologic Industries, Kibbutz Beit Haemek, Israel) that contained 1% penicillin/streptomycin (Beyotime Institute of Biotechnology, Nanjing, China) at 37 ℃ in an incubator with 21% O_2_ and 5% CO_2_, to provide normoxic conditions. Hypoxic treatment was utilised by incubator with 5% CO_2_, 1% O_2_, and 94% N_2._

### Real-time quantitative PCR (RT-qPCR)

Total RNA was obtained from cell lines using TRIzol reagent (CW Biotech, Beijing, China), cDNA was synthesized from total RNA by using the iScript cDNA synthesis kit (Bio-Rad, Hercules, CA, USA). Quantitative real-time PCR was performed by using the ABI Power SYBR Green PCR Master Mix (Applied Biosystems, Foster City, CA, USA) with the 7900 HT Sequence Detection System (Applied Biosystems). β-actin was used as internal control for gene expression. PCR primer pairs were synthesized by Sangon Biotech (Shanghai, China), and the primer sequences used were:

ISG15 forward primer-GTGGACAAATGCGACGAACC;

ISG15 reverse primer-TCGAAGGTCAGCCAGAACAG;

ZFP36 forward primer-GACTGAGCTATGTCGGACCTT;

ZFP36 reverse primer-GAGTTCCGTCTTGTATTTGGGG;

β-actin forward primer-TCCTGTGGCATCCACGAAACT;

β-actin reverse primer-GAAGCATTTGCGGTGGACGAT.

### Western blotting (WB)

Total proteins were extracted by RIPA lysis buffer (Beyotime Institute of Biotechnology), separated on SDS-PAGE gels and transferred to PVDF membranes (EMD Millipore, Billerica, MA, USA). Membranes were blocked with 5% nonfat milk in Tris-buffered saline with Tween-20, and then incubated with primary antibody overnight at 4 °C, and with corresponding secondary antibody (1:40 000; Catalogue No: SA00001-1, SA00001-2; ProteinTech, Chicago, IL, USA). The primary antibody being used are as follows: rabbit anti-human ISG15 (1:1000; Catalogue No: 15981-1-AP; ProteinTech), rabbit anti-human ZFP36 (1:1000; Catalogue No: 12737-1-AP; ProteinTech), mouse anti-human α-tublin (1:100,000; Catalogue No: 66031-1-Ig; ProteinTech). Protein bands were visualized with ECL (Beyotime Institute of Biotechnology).

### Plasmids transfection

Two types of plasmids, including plasmids overexpressing ISG15 and plasmids with knocked-down ISG15 (Genechem, Shanghai, China), were constructed. Plasmids were transfected into 22RV-1 and PC-3 with NEOFECT™ DNA transfection reagent (Neofect biotech, Beijing, China) according to protocol.

### Wound-healing assay

22RV-1 and PC-3 cell lines were seeded into 6-well plates, transfected with the indicated plasmids, and cultured to confluence on plates. The cell layer was scratched with a 200-μl pipette tip, and detached cells were removed. At least three scratched fields were photographed immediately for each sample. After an interval of 48 h, the photographs were taken again in the same fields. Cell migration was evaluated by measuring the cell-covered area.

### Transwell migration assay

22RV-1 and PC-3 cells, which were transfected with the indicated plasmids, were cultured in the chambers for 48 h and 24 h respectively. Cells were fixed in methanol for 15 min and then stained with 1 mg/ml crystal violet for 20 min. At least five fields for each chamber were photographed after staining.

## Results

### Identification of hypoxia-related differentially expressed genes (DE-HRGs) in PCa

**Fig. 1:**
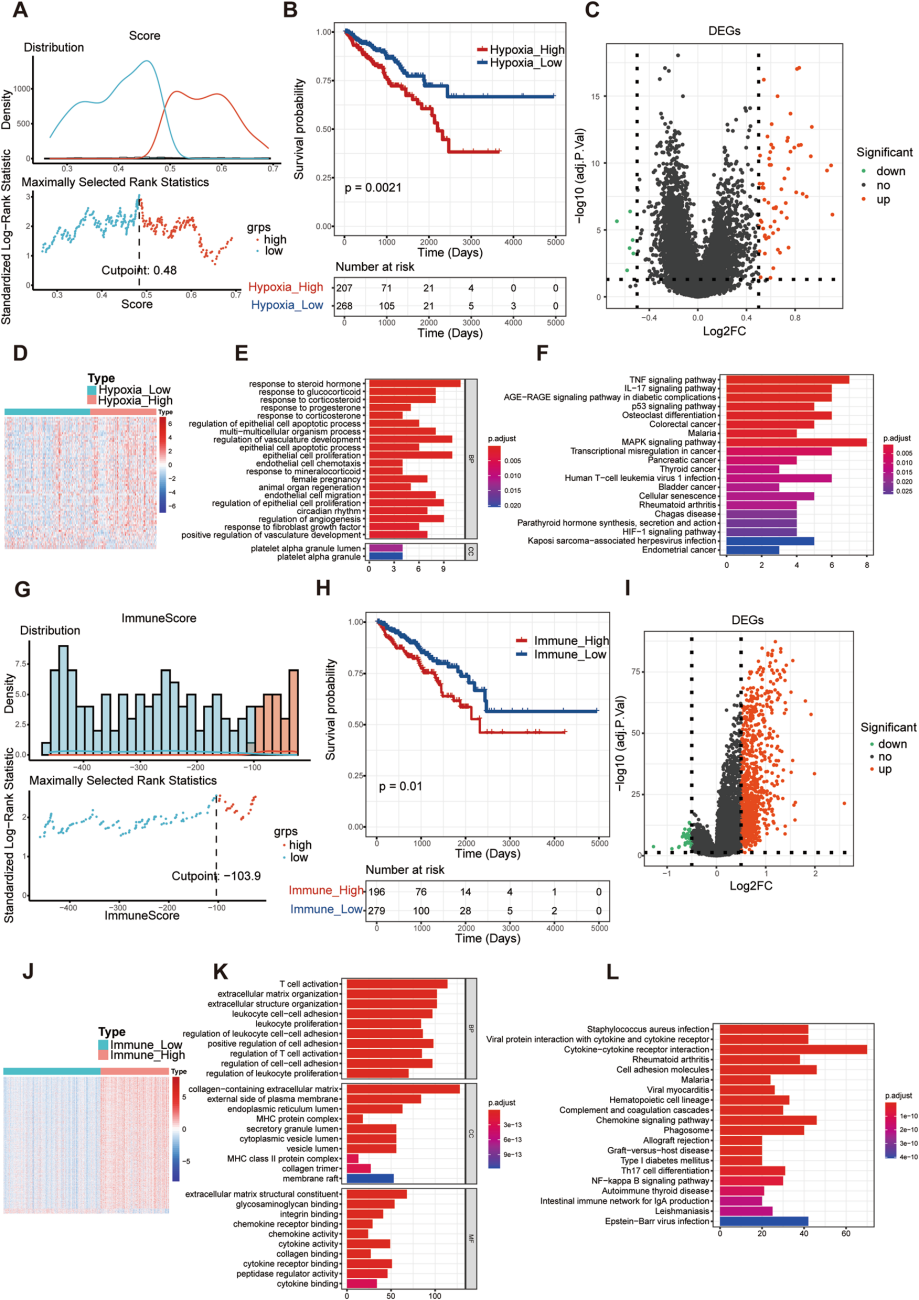
Identification of hypoxia- and immune-related DEGs (DE-HRGs and DE-IRGs) in PCa. **A** The PCa patients of TCGA database were assigned to high- and low-hypoxia score groups based on optimal cut-off value of hypoxia score. **B** Kaplan–Meier (K–M) curve for disease-free survival (DFS) between high- and low-hypoxia score groups. **C**, **D** The volcano map and heatmap of DEGs between high- and low-hypoxia score groups. **E**, **F** The functional enrichment analysis of the DEGs between high- and low-hypoxia score groups based on KEGG and GO. **G** The PCa patients of TCGA database were assigned to high- and low-immune score groups based on optimal cut-off value of immune score. **H** Kaplan–Meier (K–M) curve for disease-free survival (DFS) between high- and low-immune score groups. **I**, **J** The volcano map and heatmap of DEGs between high- and low-immune score groups. **K**, **L** The functional enrichment analysis of the DEGs between high- and low-immune score groups based on KEGG and GO

To assess the hypoxia status, we first calculated the hypoxia score of each patient with PCa using the maximally selected rank statistics method. Meanwhile, a significant correlation between hypoxia score and Gleason score was found (Additional file 9: Fig. S1A) among other clinicopathological factors (Additional file 9: Fig. S1B–D). The optimal cut-off value of hypoxia score was 0.48, which was used to assign patients with PCa from the TCGA database to high- and low-hypoxia score groups **(**Fig. 1A, Additional file 1: Table S1). As shown in Fig. 1B, patients with PCa and a high hypoxia score showed poor disease-free survival (DFS) compared to patients with a low score (P = 0.0021). To further investigate the association between gene expression and hypoxia scores, we performed a differential expression analysis between high- and low-hypoxia scores. A total of 69 DEGs, including 63 upregulated and 6 downregulated genes, were obtained (Fig. 1C and 1D, Additional file 2: Table S2). These DE-HRGs were involved in the HIF-1 signalling pathway, various cancers, and several immune-related pathways in the progression of PCa (Fig. 1E, F).

### Identification of immune-related DEGs (DE-IRGs) in PCa

Furthermore, we determined the immune scores of 475 PCa samples with complete survival data using the ESTIMATE algorithm. Consistent with hypoxia score, there was a significant correlation between immune score and Gleason Score (Additional file 10: Fig. S2A) while other clinicopathological factors showed negative results (Additional file 10: Fig. S2B–D). The optimal cut-off value of the immune score was − 103.9, which divided the PCa cases into high- and low-immune score groups (Fig. 1G, Additional file 3: Table S3). To establish a possible correlation between the immune score and DFS, we conducted a Kaplan–Meier (K–M) analysis between the high- and low-immune score groups. The results showed that a high-immune score was significantly associated with a poorer outcome (P = 0.01) (Fig. 1H). In addition, a total of 981 DEGs, including 946 upregulated genes and 35 downregulated genes, were identified between the high- and low-immune score groups (Fig. 1I and J, Additional file 4: Table S4). These DEGs were associated with immune-related biological processes and signalling pathways, such as T cell activation, MHC protein complex, chemokine receptor binding, chemokine signalling pathways, Th17 cell differentiation, and NF-kappa B signalling pathway (Fig. 1K and 1L).

### Identification of hypoxia- and immune-related DEGs for PCa

**Fig. 2:**
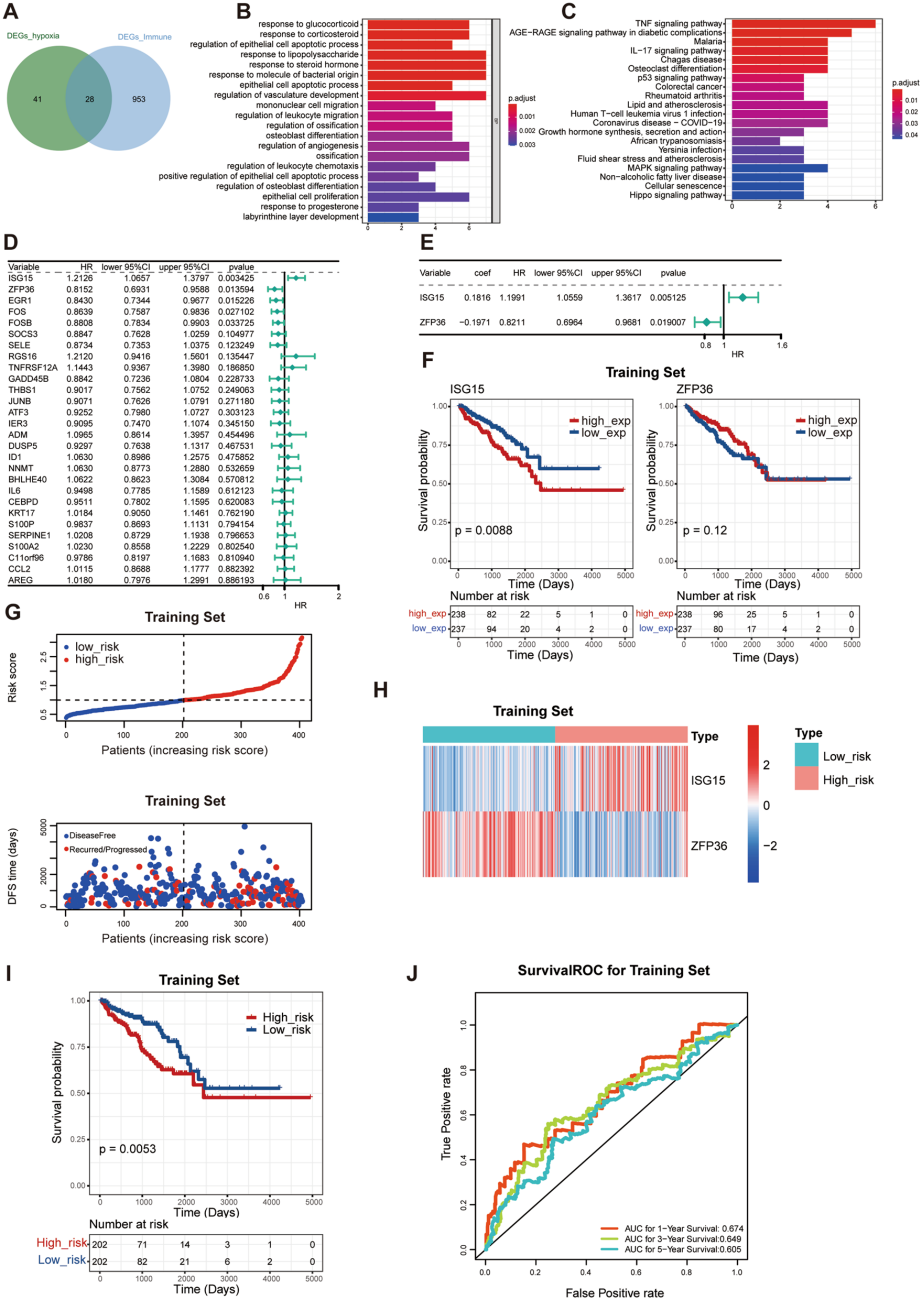
Construction and evaluation of prognostic signature based on DE-HRGs and DE-IRGs in the training set. **A** Hypoxia- and immune-related DEGs were obtained by the Venn analysis. **B**, **C** The top 20 enriched biological processes (BPs) of GO terms and KEGG pathways based on hypoxia- and immune-related DEGs. **D** Forest plot of univariate Cox analysis. **E** Forest plot of stepwise multivariate regression analysis. **F** The K–M curves of the ISG15 and ZFP36. **G**, **H** The distribution of risk scores for each patient and the expression of the two prognostic genes in high- and low- risk groups. **I** Kaplan–Meier curve of high- and low-risk groups in the training set. **J** ROC curves of 1/3/5 years predictions in training set

In total, 28 hypoxia- and immune-related DEGs were obtained using Venn analysis (Fig. 2A). To better understand the biological roles of the 28 hypoxia- and immune-related DEGs, the clusterProfilerR package was employed to perform GO annotation and KEGG pathway enrichment analyses. Figure 2B and 2C illustrate the top 20 enriched BPs in terms of GO and KEGG pathways. GO analysis revealed that these DEGs were markedly enriched in the regulation of leukocyte migration and chemotaxis, and the regulation of vasculature development. In addition, the significantly enriched pathways for these 28 DEGs were TNF signalling pathway, colorectal cancer, AGE-RAGE signalling pathway in diabetic complications, malaria, p53 signalling pathway, Chagas disease, osteoclast differentiation, and IL-17 signalling pathway.

### Development of the hypoxia- and immune-related risk signature in PCa

In the training set, five hypoxia- and immune-related genes were identified through univariate Cox regression analysis, which were significantly associated with DFS of patients with PCa (P < 0.005) (Fig. 2D, Additional file 5: Table S5). Two genes, ISG15 and ZFP36, were selected through stepwise multivariate regression analysis (Fig. 2E, Additional file 6: Table S6), and used to develop a prognostic gene signature. The K–M curves of the two hypoxia- and immune-related genes are shown in Fig. 2F.

The risk score of each patient with PCa was calculated using the formula mentioned above, and the patients were categorised into high- and low-risk groups according to the median risk score. The distribution of risk scores for each patient and the expression of the two prognostic genes are presented in Fig. 2G and 2H, respectively. Patients with PCa and a high-risk score had a shorter DFS time than those with a low-risk score (Fig. 2I) (P = 0.0053). The predictive accuracy of the two hypoxia- and immune-related signatures was assessed using time-dependent ROC curves. The area under the curve (AUC) scores of ROC curves at 1, 3, and 5-year were 0.674, 0.649, and 0.605, respectively, indicating that the prognostic signature had a good predictive performance (Fig. 2J).

### Validation of the hypoxia- and immune-related risk signature in MSKCC database

**Fig. 3:**
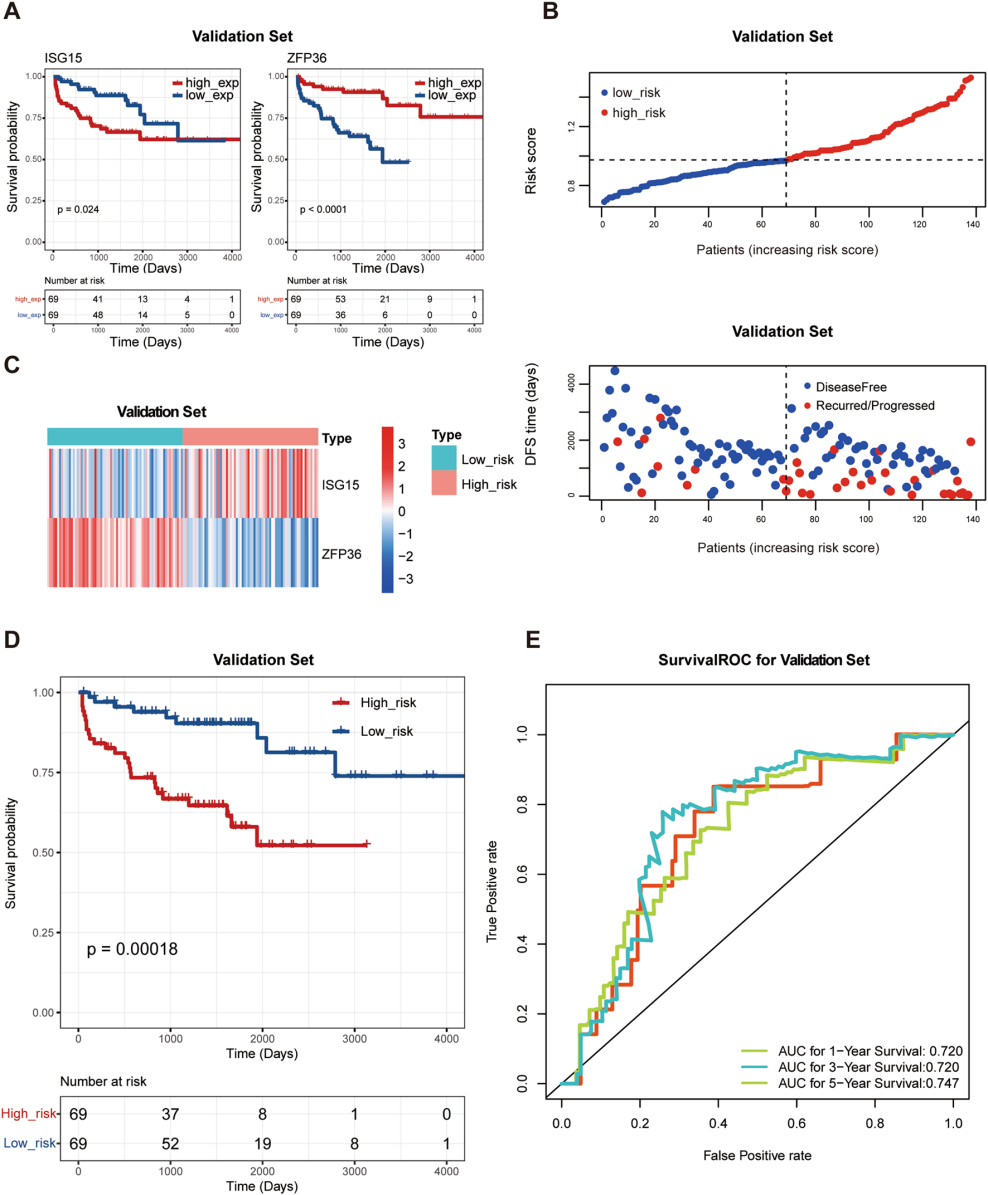
Validation of the prognostic signature in the MSKCC dataset. **A** The K–M curves of ISG15 and ZFP36 in the validation dataset. **B**, **C** The distribution of risk scores for each patient and the expression of the two prognostic genes in high- and low- risk groups in the validation set. **D** Kaplan–Meier curve of high- and low-risk groups in the validation set. **E** ROC curves of 1/3/5 years predictions in the validation set

To further demonstrate the predictive accuracy of the hypoxia- and immune-related risk signature, an independent external dataset, the Memorial Sloan Kettering Cancer Centre (MSKCC database), was applied to validate the results obtained from the training set. The K–M curves of the two hypoxia- and immune-related genes in MSKCC are shown in Fig. 3A. In the validation dataset, we divided patients with PCa into high- and low-risk groups based on the median risk score calculated with the same equation. The risk score distribution and gene expression patterns for patients in the validation set are shown in Fig. 3B, C. The patients in the high-risk group had markedly shorter DFS than those in the low-risk group (Fig. 3D,  P = 0.00018), which was consistent with the results of the training dataset. The AUCs of the hypoxia- and immune-related risk signature were 0.720, 0.720, and 0.747 at 1-, 3-, and 5-years, respectively. This further suggested a significant predictive value of the prognostic signature for clinical outcomes (Fig. 3E).

### Identification of independent prognostic indicators for PCa

**Fig. 4:**
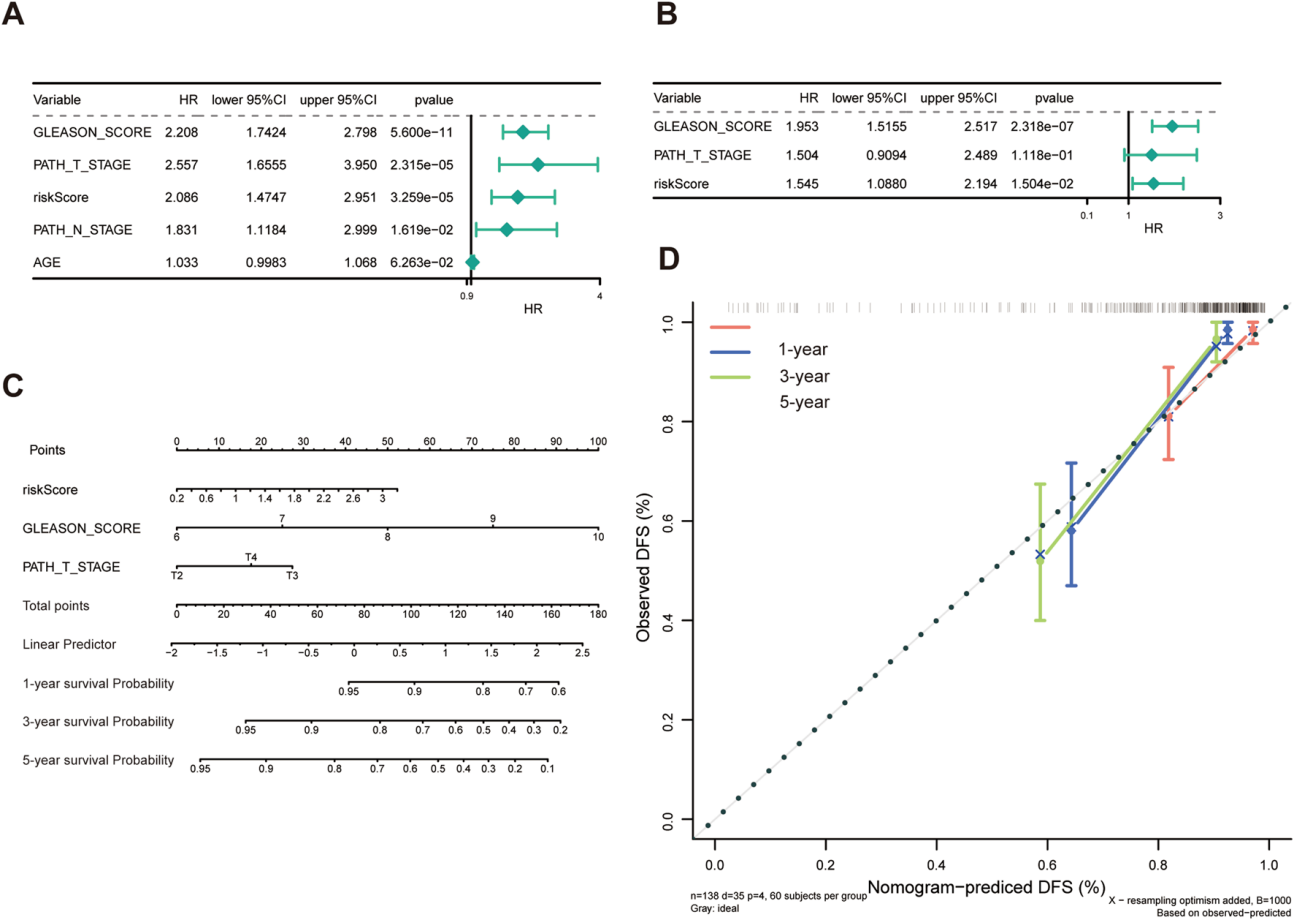
Identification of independent prognostic indicators for PCa. **A** The univariate Cox regression analysis containing risk score and clinicopathological features. **B** The multivariate Cox regression analysis to screen independent prognostic factors. **C** The nomogram predicting 1, 3, and 5 year survival rates for PCa patients. **D** 1, 3, and 5 year calibration curves of the nomogram

To further explore the relationship between the risk score and clinical outcomes, we analysed the differences in clinical features of the high- and low-risk groups in the training and validation sets, respectively. As illustrated in Tables 1 and 2, the risk score was associated with the Gleason score, T stage, and N stage of patients with PCa (P < 0.05). Based on these clinical features, univariate and multivariate Cox regression analyses were conducted to identify independent predictors of PCa prognosis. Gleason score and risk score were independent prognostic factors for PCa (Fig. 4A, B). Gleason score and risk score were integrated to generate a nomogram in the training set (Fig. 4C). In addition, the calibration curves and the nomogram showed good agreement, which provided a quantitative method for clinicians to predict the survival probability of 1-, 3-, and 5-year survival rates (Fig. 4D).

**Table 1:** The risk score was associated with the Gleason score, T stage, and N stage of patients with PCa in the training set

	Total (N = 404)	Expression		P-value
		High_risk (N = 202)	Low_risk (N = 202)	
Gleason_Score				
6	23 (5.7%)	7 (3.5%)	16 (7.9%)	0.005
7	201 (49.8%)	87 (43.1%)	114 (56.4%)	
8	57 (14.1%)	32 (15.8%)	25 (12.4%)	
9	120 (29.7%)	74 (36.6%)	46 (22.8%)	
10	3 (0.7%)	2 (1.0%)	1 (0.5%)	
Age (years)				
> = 55	62 (15.3%)	25 (12.4%)	37 (18.3%)	0.129
< 55	342 (84.7%)	177 (87.6%)	165 (81.7%)	
pT				
T2	140 (34.7%)	50 (24.8%)	90 (44.6%)	< 0.001
T3	254 (62.9%)	144 (71.3%)	110 (54.5%)	
T4	10 (2.5%)	8 (4.0%)	2 (1.0%)	
pN				
N0	330 (81.7%)	150 (74.3%)	180 (89.1%)	< 0.001
N1	74 (18.3%)	52 (25.7%)	22 (10.9%)

**Table 2:** The risk score was associated with the Gleason score of patients with PCa  in the validation set

	Total (N = 138)	Expression		P-value
		High_risk (N = 69)	Low_risk (N = 69)	
Gleason_Score				
6	41 (29.7%)	18 (26.1%)	23 (33.3%)	< 0.001
7	76 (55.1%)	32 (46.4%)	44 (63.8%)	
8	11 (8.0%)	9 (13.0%)	2 (2.9%)	
9	10 (7.2%)	10 (14.5%)	0 (0%)	
pT				
T2	86 (62.3%)	37 (53.6%)	49 (71.0%)	0.093
T3	45 (32.6%)	27 (39.1%)	18 (26.1%)	
T4	7 (5.1%)	5 (7.2%)	2 (2.9%)

### Functional enrichment analysis of prognostic hypoxia- and immune-related genes

**Fig. 5:**
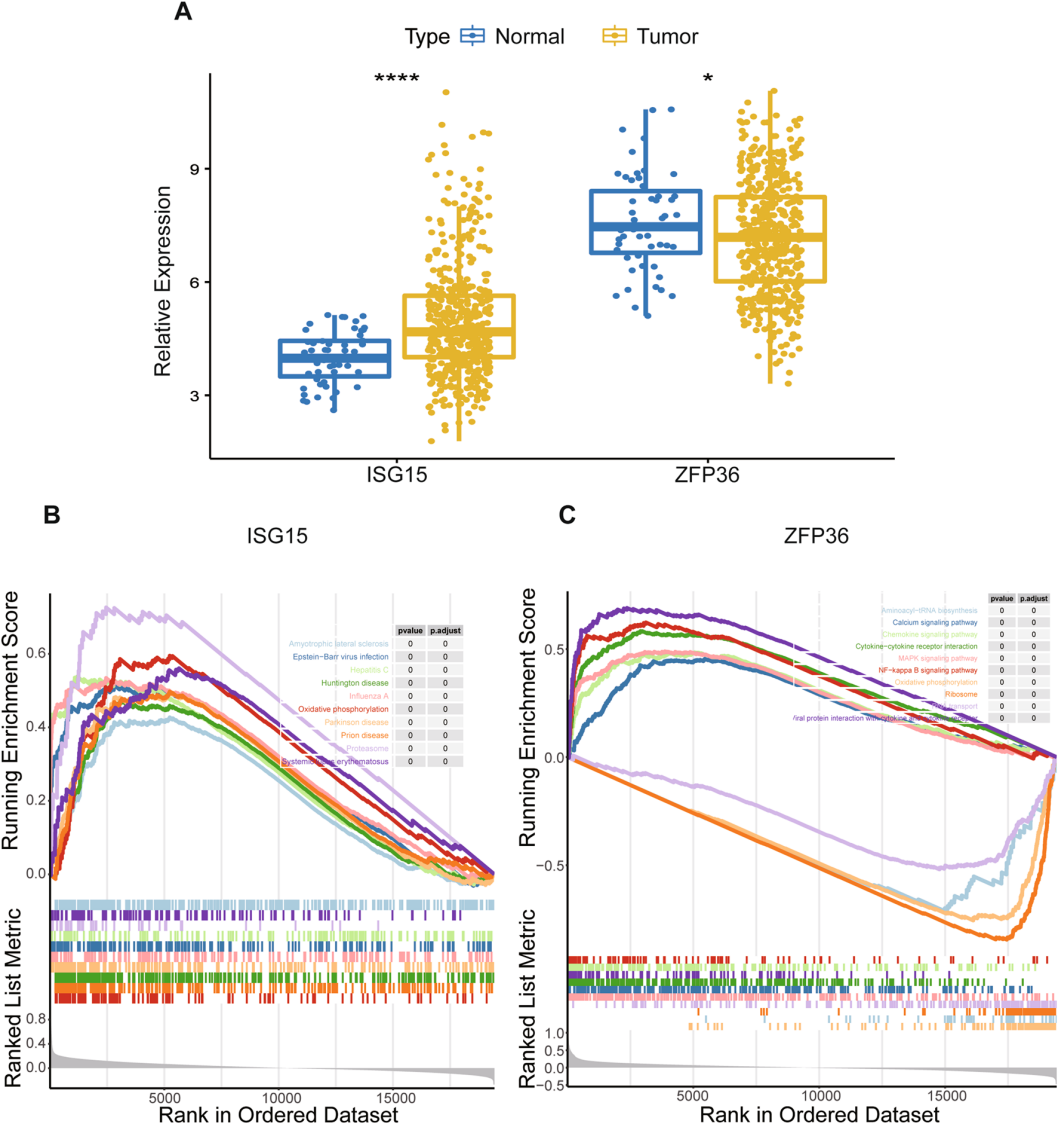
GSEA of prognostic hypoxia- and immune-related genes. **A** The differential expression of ISG15 and ZFP36 in normal and tumor tissues. **B** The top10 KEGG pathways correlated with ISG15. **C** The top10 enriched KEGG pathways correlated with ZFP36

As shown in Fig. 5A, the expression of the two prognostic hypoxia- and immune-related genes was compared between tumor tissues and normal tissues. Compared with normal tissues, the expression of ISG15 was significantly increased in tumor tissues, whereas the expression of ZFP36 was significantly decreased. To further explore the functions of the two prognostic genes in PCa progression, GSEA was employed to search for pathways enriched in TCGA samples. A total of 72 KEGG pathways were enriched, from which 46 were enriched in genes positively correlated with ISG15, and 26 were enriched in genes negatively correlated with ISG15 (Additional file 7: Table S7). The top 10 pathways are visually displayed in Fig. 5B. Amyotrophic lateral sclerosis, Epstein-Barr virus infection, hepatitis C, Huntington’s disease, influenza A, oxidative phosphorylation, Parkinson’s disease, prion disease, proteasome, and systemic lupus erythematosus were differentially enriched in ISG15 high expression samples. In addition, 197 KEGG pathways were significantly enriched, from which 151 were enriched in genes that were positively related to ZFP36, and 46 were enriched in genes that were negatively related to ZFP36 (Additional file 8: Table S8). Figure 5C illustrates that upregulation of ZFP36 was associated with viral protein interaction with cytokine and cytokine receptor, NF-kappa B signalling pathway, cytokine-cytokine receptor interaction, MAPK signalling pathway, chemokine signalling pathway, and calcium signalling pathway. However, downregulation of ZFP36 was correlated with RNA transport, aminoacyl-tRNA biosynthesis, oxidative phosphorylation, and ribosomes.

### The risk score was associated with immune cell infiltration

**Fig. 6:**
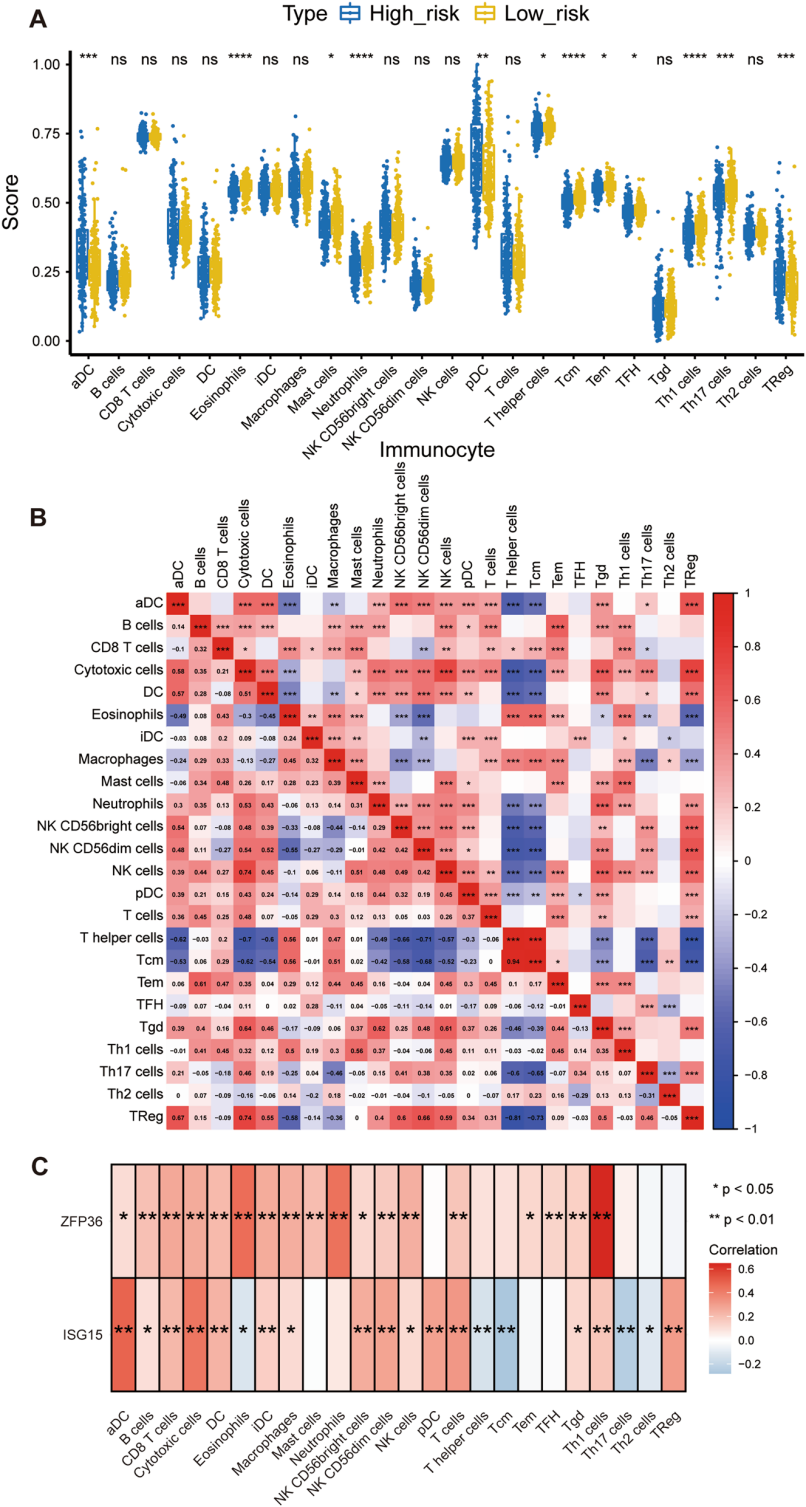
The risk score and prognostic genes were associated with immune cell infiltration. **A** Differential fractions of 24 immune cells in low- and high-risk groups. **B** The correlation between 24 immune cells. **C** The Pearson correlation between 24 immune cells and prognostic genes

The immune cell infiltration level of each PCa sample in the TCGA database was evaluated using the ssGSEA algorithm. The enrichment score of 24 immune-related gene sets for each PCa sample was quantified to estimate the abundance of immune cells in the tumor immune microenvironment. Subsequently, we analysed the differences in immune infiltration levels between the high- and low-risk groups. The infiltration levels of eosinophils, neutrophils, Tcm, Tem, TFH, Th1 cells, and Th17 cells were significantly lower in the high-risk group than in the low-risk group. Conversely, aDC, pDC, T helper cells, and Treg cells were significantly higher in the high-risk group (Fig. 6A) (P < 0.05). In general, the results indicated that, to some extent, the risk score was associated with immune cell infiltration in PCa, which may affect cancer progression positively or negatively. Moreover, the relationships above were complex, which could not be too absolute, and they were mentioned in our discussion below. The correlation between the 24 immune cells is shown in Fig. 6B.

To further explore the relationship between the two prognostic hypoxia- and immune-related genes and the infiltration of immune cells, we conducted Pearson’s correlation test to calculate and analyze the correlation coefficients of the two genes in the 24 immune-related subsets (Fig. 6C). We found that the two genes were closely correlated with the tumor-infiltrating immune cell subset, revealing that the prognostic genes mainly participate in the immune response during PCa progression (P < 0.05).

### Expression of ISG15 and ZFP36 is increased and decreased in PCa respectively and ISG15 is positively correlated with hypoxia

**Fig. 7:**
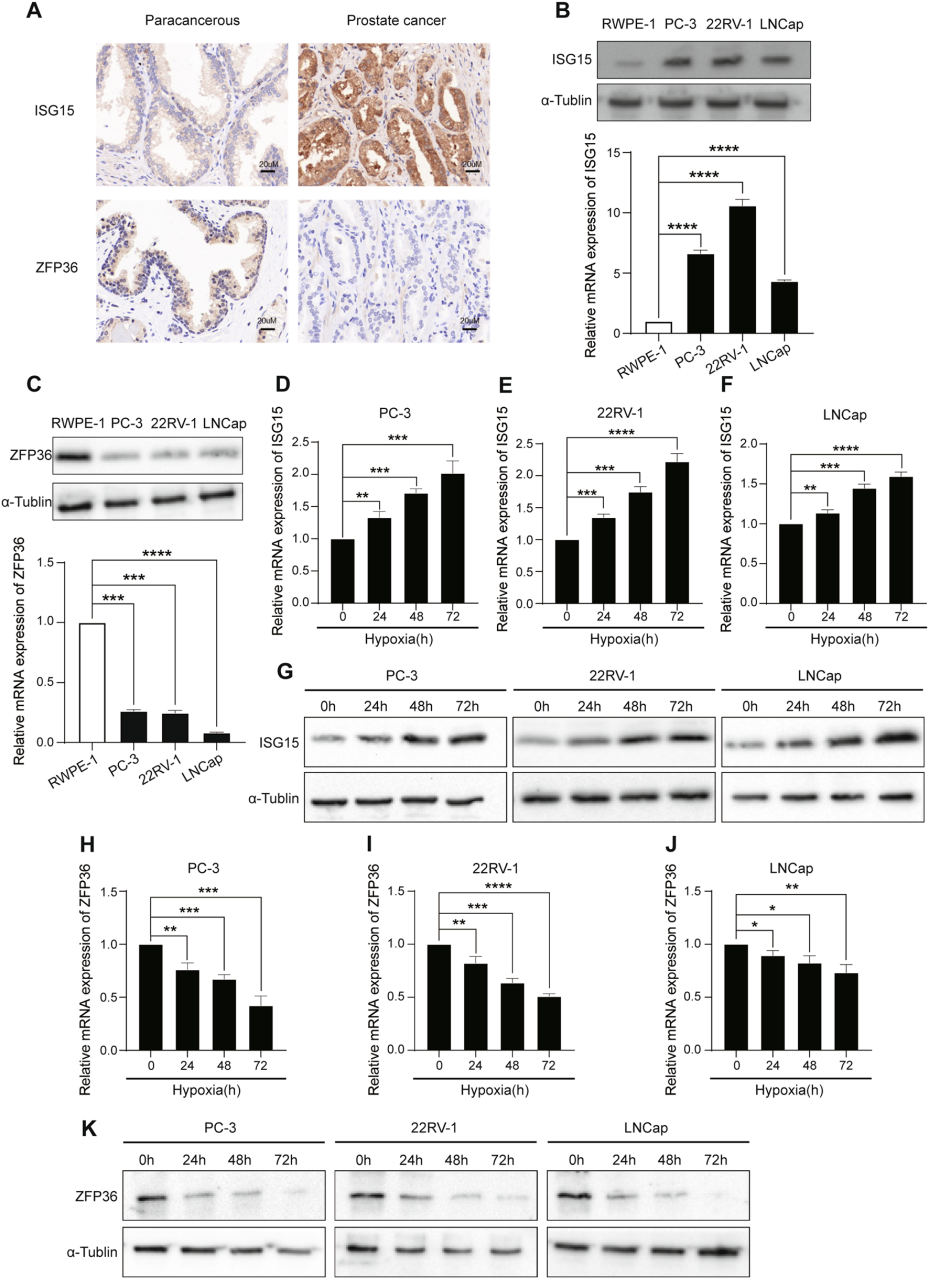
Expression of ISG15 and ZFP36 is increased and decreased in PCa respectively and is correlated with hypoxia. **A** The expression of ISG15 and ZFP36 increased and decreased respectively in PCa than in paracancerous tissues. **B**, **C** The expression of ISG15 and ZFP36 were significantly higher and lower respectively in PC-3, 22RV-1, LNCaP than in RWPE-1 at transcription level and protein level. **D**–**K** The expression level of ISG15 and ZFP36 in PC-3,22RV-1 and LNCaP was increased and decreased by the hypoxia at transcription level and protein level. **P < 0.01, ***P < 0.001, ****P < 0.0001 vs. RWPE-1 and 0 h

We demonstrated the differential expression of ISG15 and ZFP36 in PCa and paracancerous tissue by immunohistochemical staining and western blot (WB) and in PCa cell lines and normal prostate epithelial cell lines by real-time quantitative PCR (RT-qPCR) and WB. Immunohistochemical and WB results of PCa and paracancerous tissues showed differences in the expression of ISG15 and ZFP36, respectively (Fig. 7A and Additional file 11: Fig. S3). As shown in Fig. 7B, C, the expression level of ISG15 was significantly higher in PC-3, 22RV-1, and LNCaP than in RWPE-1. ZFP36 showed opposite results. After hypoxic treatment of 22RV-1, PC-3, and LNCaP cells for 24 h, 48 h, 72 h, respectively, we performed RT-qPCR and WB to demonstrate that the expression level of ISG15 and ZFP36 was positively and negatively correlated with hypoxia, respectively. (Fig. 7D–K).

### Overexpression of ISG15 promote migration and invasion ability of PCa cells

**Fig. 8:**
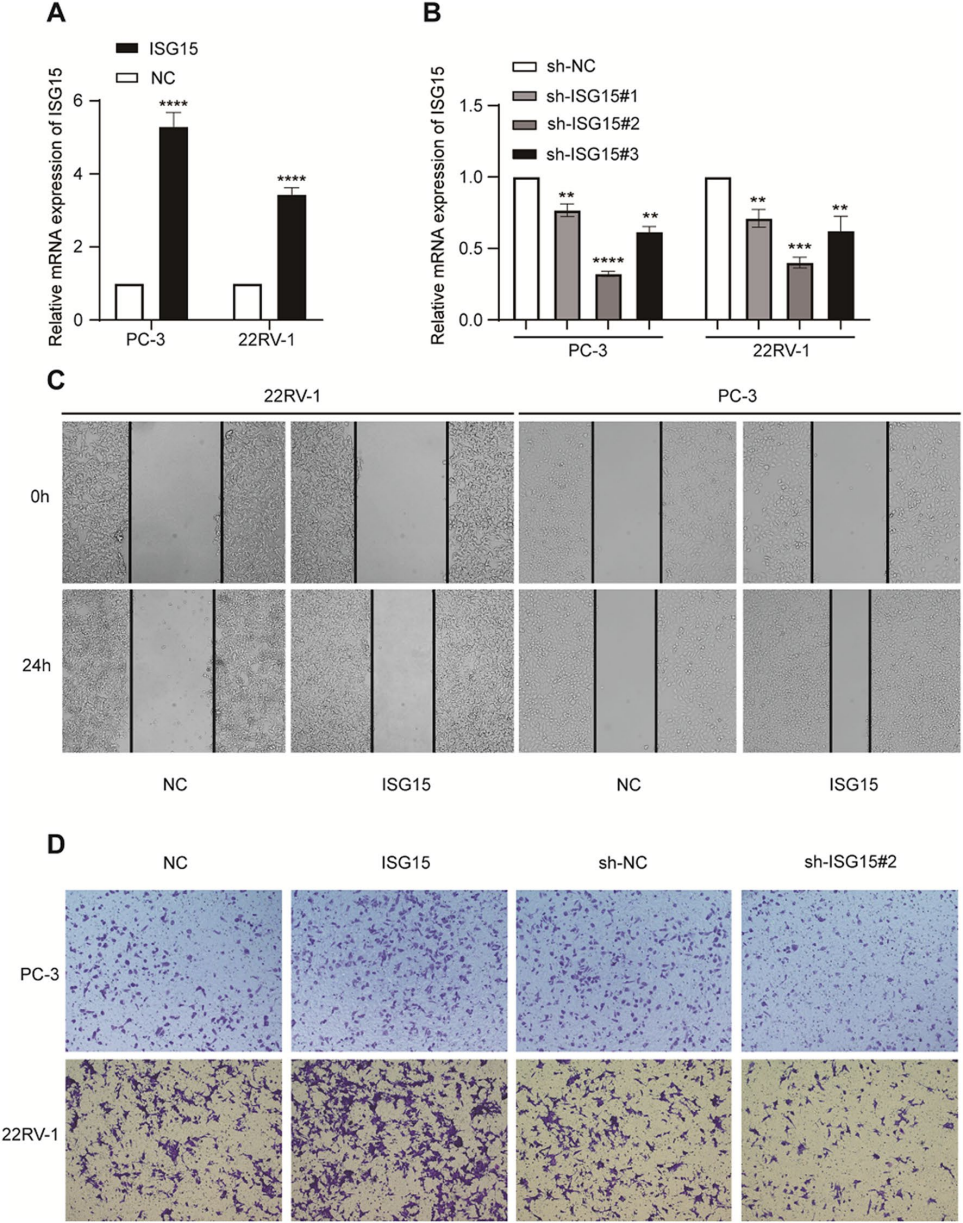
Overexpression of ISG15 promote migration and invasion ability of PCa cells. **A**, **B** Relative mRNA expression of ISG15 in PC-3 and 22RV-1 transfected with plasmids ISG15, NC, sh-ISG15, and sh-NC. **C** Overexpression of ISG15 promoted migration of PC-3 and 22RV-1. **D** Overexpression and knockdown of ISG15 promoted and inhibited, respectively, invasion ability of PC-3 and 22RV-1. **P < 0.01, ***P < 0.001,****P < 0.0001

After verifying that ISG15 expression levels were positively regulated by hypoxia, we performed wound-healing and transwell invasion assays using 22RV-1 and PC-3 cells after transfection (Fig. 8A–D). The results showed that a high expression of ISG15 could promote the migration and invasion ability of PCa cells, thereby suggesting that high expression of ISG15, under hypoxic conditions, may promote poor prognosis of PCa.

## Discussion

PCa is the second most prevalent cancer in men worldwide, with the incidence and mortality rates increasing every year. Advanced PCa has not only an aggressive and metastatic cellular phenotype, but also a propensity for drug resistance and an escape from immune destruction [1, 2, 22]. In recent years, a growing number of studies have reported that hypoxia can induce immune escape in PCa cells [22–24]. Kazantseva et al. expounded that hypoxia stimulated elevated 133TP53 gene expression in PCa cells, resulting in an immunosuppressive infiltrate [24]. Chen et al. reported that HIF-1 affected the cytotoxicity of NK cells against PCa by upregulating miR-224 to inhibit the NCR1/NKp46 pathway, thereby inducing immune escape of tumor cells [23].

In our study, we screened for a combined genetic marker associated with hypoxia and immunity to identify and assess the prognosis of PCa patients. Among the marker genes, ISG15 and ZFP36 were selected as predictors of prognosis in patients with PCa. They can promote and inhibit the aggressiveness of tumor cells, thus affecting the prognosis of patients. Moreover, they can also be involved in the immune response of PCa and influence the infiltration of immune cells.

Based on the hypoxia score, we performed differential expression analysis between high- and low-hypoxia score groups and obtained 63 upregulated genes and 6 downregulated genes. In addition, we identified 946 upregulated genes and 35 downregulated genes between the high and low immune score groups, according to the immune score. After overlapping two sets of genes, 28 DEGs were identified. We performed GO annotation and KEGG pathway enrichment analyses to better understand their biological roles. The immune-related pathways in which these 28 DEGs were significantly enriched, were the TNF signalling pathway, the p53 signalling pathway, and the IL-17 signalling pathway. Previous studies have also reported the regulation of related molecules under hypoxic conditions, further involving immune-related pathways [17, 23, 24]. Kazantseva et al. proved that 133p53, one of the isoforms of p53 that lacks the N terminus and is alternatively spliced at the C terminus, could regulate genes involved in immune signalling that have a direct bearing on immune cell activity and recruitment under anoxic conditions. Gene enrichment analysis of cancers also showed that133p53 was associated with pathways involved in immune signalling [24]. Another study showed that hypoxia upregulates HIF-1, and suppresses the NCR1/NKp46 pathway by upregulating miR-224, which affects the killing capability of NK cells in prostate cancer; thus, inducing immune escape of tumor cells [23]. In addition, other researchers have demonstrated that activation of the JAK1, 2/PD-L1 and Stat3/PD-L1 signalling pathways may decrease the immune cytolytic activity of NK cells toward hypoxia-induced castration-resistant prostate cancer (CRPC) cells, which is expected to provide unique ideas and targets for the immunotherapy of CRPC [17]. Through univariate Cox regression analysis and stepwise multivariate regression analysis, ISG15 and ZFP36, which have increased and decreased expression in PCa respectively, were screened out from the 28 DEGs to be prognostic genes. According to GSEA from the TCGA samples, pathways associated with ISG15 and ZFP36 were enriched, which contributes to research on the molecular mechanisms of PCa.

ISG15, also known as interferon-stimulated gene 15, has been reported to be overexpressed in PCa and to promote tumor cell proliferation in previous studies [25–27]. In our study, in addition to the high expression of ISG15 in PCa, we showed that hypoxic conditions upregulated ISG15 expression, which in turn promoted the migration and invasion of PCa cells. This causes poor prognosis in patients with PCa. It may represent a unique cancer marker with prognostic significance, may be helpful in selecting patients, and to predict the response to treatment [28].

ISG15 can trigger an increase in androgen receptor (AR) expression through androgen-mediated effects, thereby promoting the proliferation of PCa cells [26]. Moreover, specific miRNAs can regulate the expression of ISG15 through certain signalling pathways in PCa. For instance, miR-2909, encoded by the AATF genome, upregulated ISG15 expression via STAT1 signalling through the negative regulation of SOCS3 [25].

ISG15, secreted by M2 macrophages or alternatively activated macrophages, which are known to promote tumorigenesis and suppress adaptive immunity, has been implicated in the activation and phenotype modulation of immune cells and are involved in multiple tumors. It has been shown to promote the tumorigenicity of cancer stem cells and tumor immune escape [29–33].

However, to date, no studies have reported the association of ISG15 with hypoxia and immunity in PCa.

ZFP36, also known as zinc finger protein 36, the protein expression in PCa tissues was significantly lower than that in non-cancerous prostate tissues. Its upregulation in PCa was significantly associated with a low Gleason score, negative metastasis, favorable overall survival, and negative biochemical recurrence [34]. A hypothesis was put forward that zinc finger proteins may have an influential relationship with AR and telomere, which form a triad that controls the pattern of gene expression during the progression of prostate cancer [35]. Low expression of ZFP36 is associated with castration resistance in PCa [36]. The mechanism by which ZFP36 inhibits PCa progression has been previously reported [37, 38]. The promoter region of ZFP36 was bound by EGR3 and transcriptionally activated, thereby exerting a tumor-suppressive effect [38]. However, ZFP36 was also predicted as a promising upstream inhibitor of the NF-κB pathway with a role in reversing the growth of PCa [37].

Based on the level of immune cell infiltration in each PCa sample assessed by the ssGSEA algorithm, we demonstrated that the risk signature based on the composition of hypoxia- and immune-related genes, was associated with immune infiltrating cells. According to the Pearson correlation analysis, the two DEGs, including ISG15 and ZFP36, were closely correlated with tumor-infiltrating immune cell subsets, suggesting the involvement of prognostic genes in the immune response in PCa progression. It is also worth noting that infiltration of immune cells had a two-sided impact on the prognosis of patients. High infiltration of some immune cells, such as Treg cells, in a wide range of cancers may produce worse prognostic outcomes [39, 40]. This may be due to the fact that immune cells, together with immunosuppressive molecules and cytokines, form an immunosuppressive network that inhibits effective antitumor immunity [39, 41].

Chen et al. reported that chronic infection with Epstein-Barr virus may lead to upregulated expression and release of ISG15 in nasopharyngeal carcinoma cells, which promotes the formation of a macrophage M2 phenotype, further inhibiting the anti-tumor CD8+ T cell response [42]. Additionally, ISG15 can also trigger the activation of CD8+ T cells through the mediation of NK cells, which then elevates its increase in numbers, thereby inhibiting the progression of ovarian cancer [43]. As for ZFP36, Long et al. demonstrated that androgen deprivation therapy (ADT) can remodel the tumor immune microenvironment in PCa. Furthermore, ZFP36, the immune-related gene, may play a vital role in the ADT immune remodelling process, thus resulting in differences in PSA recurrence-free survival and immune infiltration [36].

Clinically, hypoxia and immune responses are closely related to various cancer treatments. Under hypoxic conditions, cancer cells become resistant to chemotherapy and radiotherapy, and develop immune escape [44]. It is well known that the success of these therapies depends on normal oxygen levels [44]. Therefore, we need to explore more effective treatments to overcome the suppression of hypoxia and its impact on the immune response.

In recent years, a variety of approaches have been developed. Targeting hypoxia to enhance the efficacy of immunotherapy is underway [45]. Unlike previous immunotherapies that did not consider the hypoxic tumor microenvironment, researchers have begun to explore tumor reoxygenation, hypoxia-activated prodrugs, targeting of the HIF-1 pathway, hypoxia-targeted biologicals, and metabolic intervention [46, 47]. In addition to the aspects mentioned above, there were also some studies that offered different perspectives on the treatment of hypoxic tumor microenvironment. Several studies have shown that increasing the partial pressure of oxygen in tumor is effective in improving the prognosis [48–50]. Therefore, maintaining the oxygen supply inside the tumor can help with the treatment. Two pharmaceutical technologies that belong to acellular oxygen therapeutic classes, including Hgb-based oxygen carriers (HBOCs) and perfluorocarbon emulsions (PFCs), have been investigated for a wide range of ischaemia applications [44]. The PFCs work like an intravenous hyperbaric system, and HBOCs bind and chemically release oxygen. They act as the leading candidates of acellular oxygen therapeutic classes [51, 52]. In addition, 18F-FMISO PET imaging was also applied in preclinical models of breast and colon cancer to probe the tumor and its surrounding microenvironment before and during PD-1 and CTLA-4 checkpoint blockade to quantify tumor hypoxia. Hypoxic signals from PET imaging are used to add hypoxia-targeted therapy to unresponsive tumor, which ultimately provides therapeutic synergy [53].

In this study, we screened for and predicted hypoxia- and immune-related DEGs that were relevant to the prognosis of PCa patients and immune cell infiltration. However, the modality of carcinogenic effects of the prognostic genes and the mechanisms of interaction between prognostic genes and immune cells under hypoxic conditions are unclear and require further exploration.

## Conclusions

We obtained two hypoxia- and immune-related genes, ISG15 and ZFP36, which are highly and lowly expressed in PCa, respectively. Our results indicate that they are strongly associated with the prognosis of PCa patients and clearly influence the infiltration of immune cells in the tumor microenvironment. This provides a unique therapeutic target for PCa under hypoxic conditions and offers new ideas for immunotherapy.

## Supplementary Information

**Additional file 1: Table S1.**  High- and low-hypoxia score groups with PCa.

**Additional file 2: Table S2.** The DEGs between high- and low-hypoxia score groups.

**Additional file 3: Table S3.** High- and low-immune score groups with PCa.

**Additional file 4: Table S4.** The DEGs between high- and low-immune score groups.

**Additional file 5: Table S5.** Hypoxia- and immune-related genes identified by univariate Cox regression analysis.

**Additional file 6: Table S6.** Two genes, ISG15 and ZFP36, selected by stepwise multivariate regression analysis.

**Additional file 7: Table S7.**72 enriched KEGG pathways correlated with ISG15.

**Additional file 8: Table S8.** 197 enriched KEGG pathways correlated with ZFP36.

**Additional file 9: Figure S1**. Correlation between hypoxia score and clinicopathological parameters. (A) A significant correlation between hypoxia score and Gleason score. (B–D) Negative results of age, pathological T stage and pathological N stage.

**Additional file 10: Figure S2**. Correlation between immune score and clinicopathological parameters. A significant correlation between immune score and Gleason score. (B–D) Negative results of age, pathological T stage and pathological N stage.

**Additional file 11: Figure S3**. Differential expression of ISG15 and ZFP36 in PCa and paracancerous tissues. (A-B) The expression of ISG15 increased in PCa than in paracancerous tissues. (C-D) The expression of ZFP36 decreased in PCa than in paracancerous tissues.

## Data Availability

The datasets used and/or analyzed during the current study are available from the corresponding author on reasonable request.
