# Supplementary figures and images for: Identification of ISG15 and ZFP36 as novel hypoxia- and immune-related gene signatures contributing to a new perspective for the treatment of prostate cancer by bioinformatics and experimental verification
Source: J Transl Med. 2022 May 10;20:202. doi: 10.1186/s12967-022-03398-4 (PMC9092714; doi:10.1186/s12967-022-03398-4)

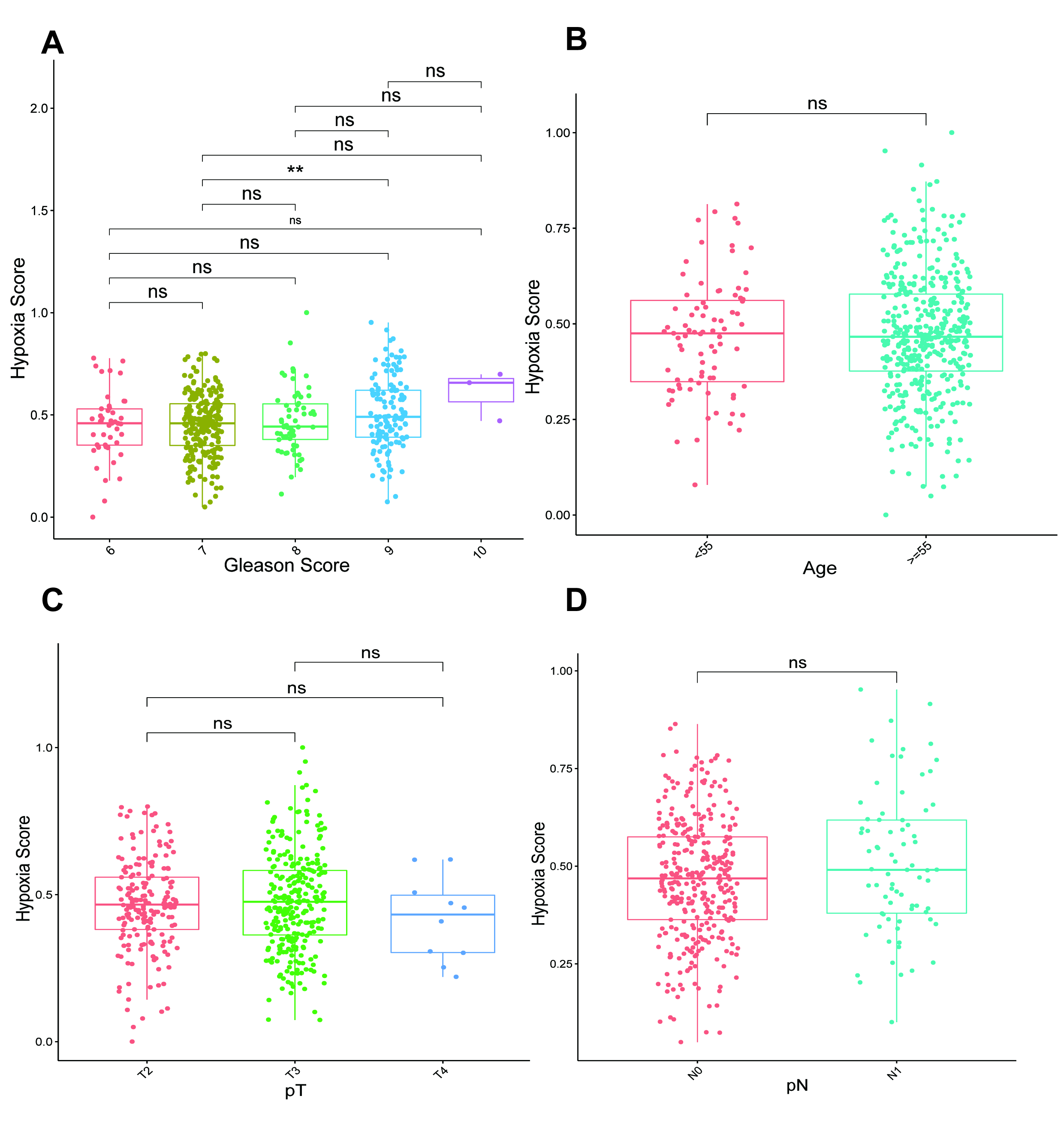

Supplement: Supplementary file 9 — Additional file 9: Figure S1. Correlation between hypoxia score and clinicopathological parameters. (A) A significant correlation between hypoxia score and Gleason score. (B–D) Negative results of age, pathological T stage and pathological N stage. [file 12967_2022_3398_MOESM9_ESM.tif]

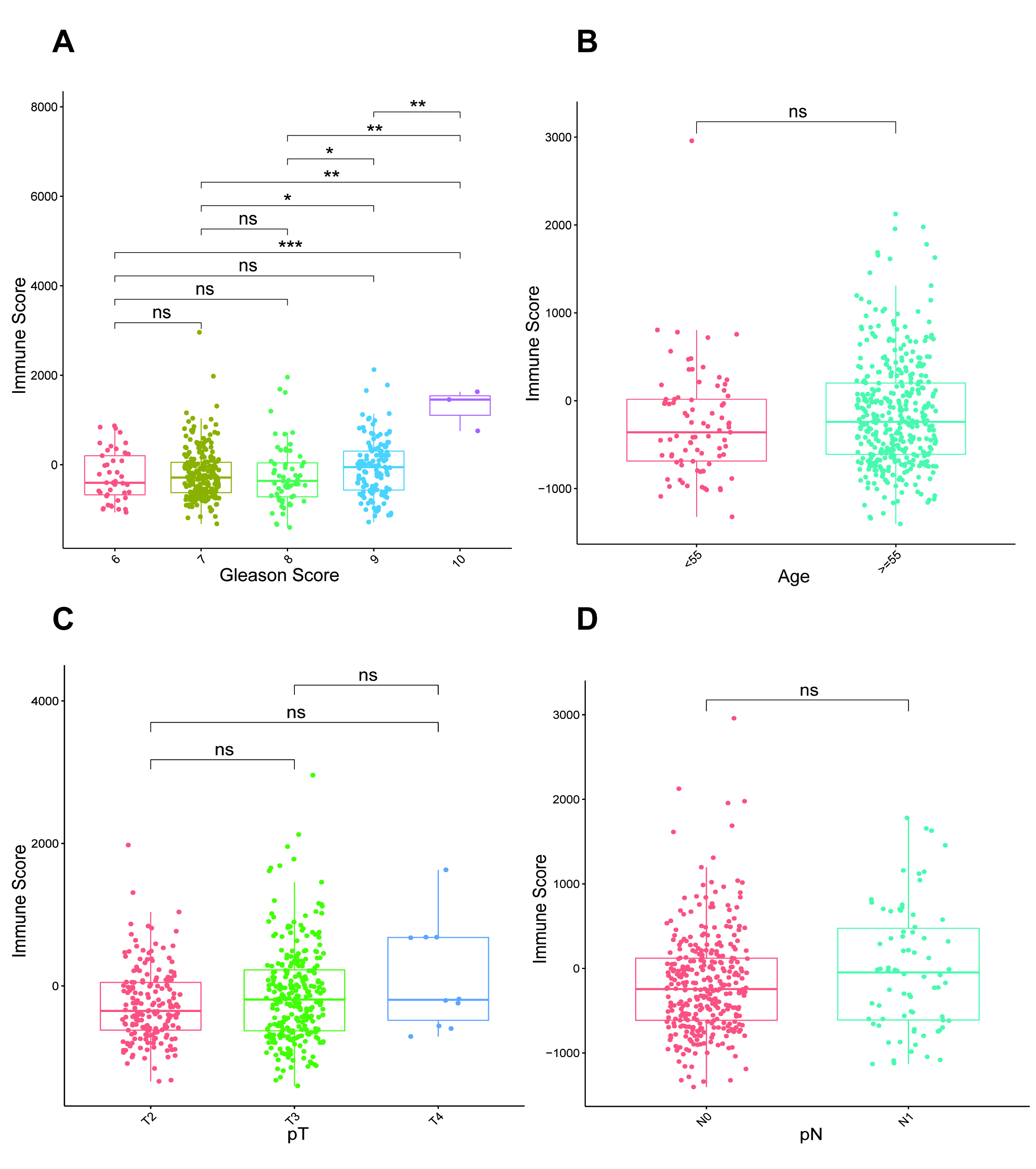

Supplement: Supplementary file 10 — Additional file 10: Figure S2. Correlation between immune score and clinicopathological parameters. A significant correlation between immune score and Gleason score. (B–D) Negative results of age, pathological T stage and pathological N stage. [file 12967_2022_3398_MOESM10_ESM.tif]

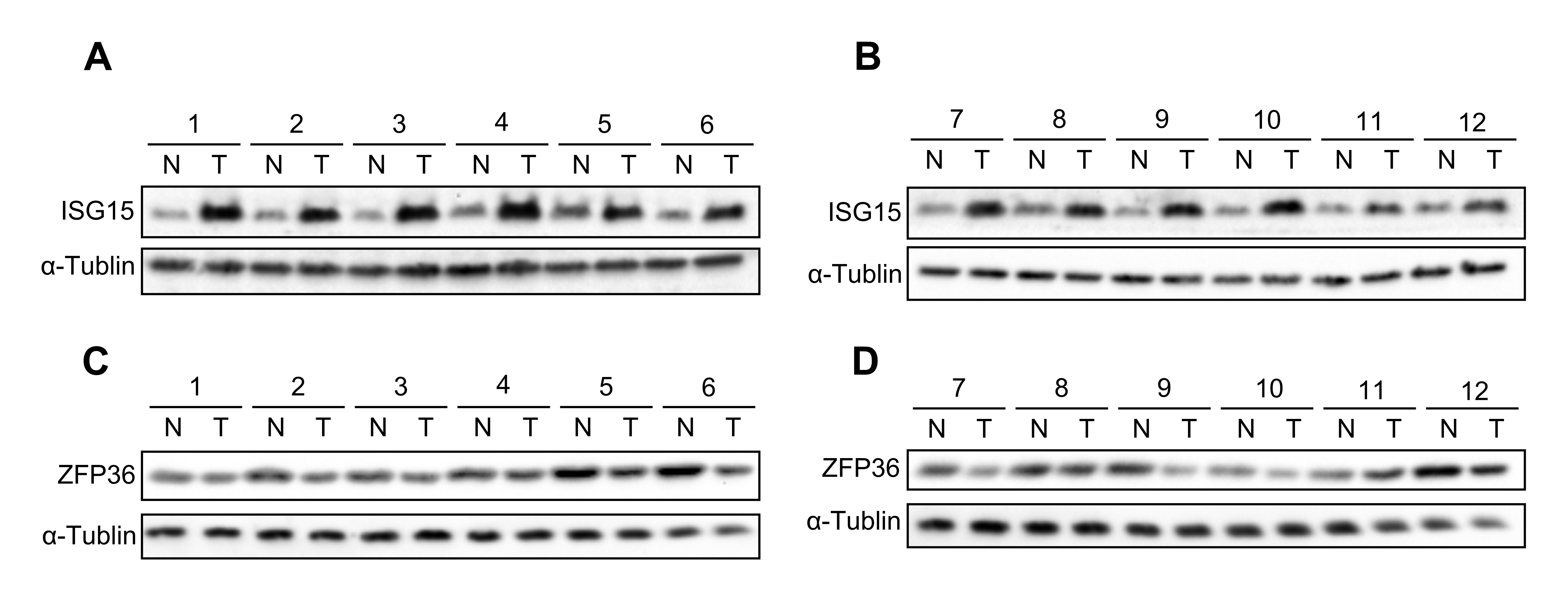

Supplement: Supplementary file 11 — Additional file 11: Figure S3. Differential expression of ISG15 and ZFP36 in PCa and paracancerous tissues. (A-B) The expression of ISG15 increased in PCa than in paracancerous tissues. (C-D) The expression of ZFP36 decreased in PCa than in paracancerous tissues. [file 12967_2022_3398_MOESM11_ESM.tif]
